# Successful Reintroduction of Golimumab in a Patient With Rheumatoid Arthritis and Prolonged Epstein-Barr Virus Reactivation With Persistent Anti-Viral Capsid Antigen IgM Antibodies: A Case Report

**DOI:** 10.7759/cureus.100683

**Published:** 2026-01-03

**Authors:** Daiki Fujimori, Haeru Hayashi, Koichiro Tahara, Hirofumi Shoda, Tetsuji Sawada

**Affiliations:** 1 Rheumatology, Tokyo Medical University Hospital, Tokyo, JPN

**Keywords:** anti-tnf-α antibody, anti–vca igm antibodies, atypical lymphocytes, epstein–barr virus, reactivation, reintroduction, rheumatoid arthritis

## Abstract

We report the case of a 63-year-old male patient with rheumatoid arthritis (RA) who experienced Epstein-Barr virus (EBV) reactivation during treatment with methotrexate (MTX) and anti-tumor necrosis factor-alpha (TNF-α) antibody, golimumab (GLM). He presented with marked atypical lymphocytosis, elevated EBV-DNA (4.90 log IU/mL [lower limit of detection: 1.60]), and persistent anti-viral capsid antigen (VCA) IgM antibodies. Discontinuation of MTX and GLM led to rapid symptom resolution, although EBV-DNA and anti-VCA IgM antibodies remained detectable. Two months later, RA relapsed and was managed with prednisolone, bucillamine, and salazosulfapyridine, but disease activity persisted. GLM was reintroduced one year after EBV reactivation, resulting in sustained remission without EBV-related complications, despite continued positivity for viral markers. This case highlights the potential feasibility of resuming anti-TNF-α antibody therapy in patients with RA exhibiting prolonged EBV reactivation and sustained IgM seropositivity under close monitoring.

## Introduction

Rheumatoid arthritis (RA) is a chronic autoimmune disease primarily affecting the synovium [1]. The treatment of RA often includes immunosuppressive agents such as methotrexate (MTX), as well as biologic and targeted synthetic disease-modifying antirheumatic drugs (DMARDs), including tumor necrosis factor-alpha (TNF-α) inhibitors, interleukin-6 (IL-6) receptor antagonists, T cell co-stimulation modulators, and Janus kinase (JAK) inhibitors. Although effective in controlling disease activity, these therapies may increase the risk of serious immune-related complications, including infections and lymphoproliferative disorders (LPD). Among these, other iatrogenic immunodeficiency-associated LPDs (OIIA-LPDs), such as MTX-associated LPD (MTX-LPD), have gained clinical attention [2]. Epstein-Barr virus (EBV) is thought to contribute to the pathogenesis of MTX-LPD [3]. A recent study by Hoshida et al. suggested that, in RA-associated OIIA-LPD, MTX combined with TNF inhibitors may increase the risk of EBV-associated LPD by promoting the clonal expansion of EBV-infected cells [4].

EBV is a ubiquitous herpesvirus that establishes latency in B cells [5]. Most individuals are infected during childhood or adolescence, after which the virus remains latent in memory B cells. Under immunosuppressive conditions, such as those following hematopoietic cell transplantation (HCT) or solid organ transplantation (SOT), EBV may reactivate, leading to chronic high EBV loads (CHEBV), which can progress to post-transplant LPD [6-9]. EBV reactivation has been increasingly recognized in patients with RA, particularly in those receiving immunosuppressive therapy [10-13]. However, cases presenting with infectious mononucleosis-like symptoms, including marked atypical lymphocytosis and generalized lymphadenopathy, remain uncommon. Clinical guidelines for managing such cases remain undefined, especially when EBV-DNA elevation and anti-viral capsid antigen (VCA) IgM positivity persist. These cases require careful differentiation from OIIA-LPD and chronic active EBV infection (CAEBV), and therapeutic decisions, particularly regarding the reintroduction of immunosuppressive agents, can be challenging. Reports documenting successful reintroduction of biologic DMARDs following prolonged EBV reactivation are scarce, and safety data remain limited.

We present a rare case of RA in which EBV reactivation occurred during MTX and golimumab (GLM) therapy, resulting in sustained EBV-DNA elevation and prolonged anti-VCA IgM positivity. The patient tolerated GLM reintroduction without clinical relapse. We describe the clinical course in detail and discuss differential diagnoses and appropriate monitoring strategies.

## Case presentation

A 63-year-old male patient with a two-year history of RA had maintained clinical remission under treatment with MTX and GLM. Initially, his RA manifested as palindromic arthralgia affecting the hands, knees, and hips. After one year, he developed persistent polyarthritis, and RA was diagnosed based on positive rheumatoid factor and anti-cyclic citrullinated peptide (CCP) antibody results. Treatment began with MTX at 8 mg/week, later increased to 10 mg/week, and prednisolone (PSL) at 5 mg/day, followed by subcutaneous GLM at 50 mg/month, which induced clinical remission. PSL was tapered off, and remission was maintained with MTX and GLM.

Two weeks before the presentation, he developed a fever up to 38°C, along with night sweats and polyarthralgia. Physical examination revealed lymphadenopathy in the cervical, axillary, and inguinal regions, without chest or abdominal abnormalities. Vital signs were stable and not clinically concerning. Laboratory findings at the time of EBV reactivation are summarized in Table 1

**Table 1:** Laboratory values in a male patient with rheumatoid arthritis who developed symptomatic Epstein–Barr virus reactivation while in clinical remission during weekly methotrexate and monthly golimumab therapy. CBC: complete blood count; WBC: white blood cell; RBC: red blood cell; PT–INR: prothrombin time–international normalized ratio; APTT: activated partial thromboplastin time; sec: seconds; AST: aspartate aminotransferase; ALT: alanine aminotransferase; LDH: lactate dehydrogenase; gGTP: gglutamyl transpeptidase; BUN: blood urea nitrogen; CK: creatine kinase; ESR: erythrocyte sedimentation rate; CRP: C-reactive protein; HPF: high-power field; IgG: immunoglobulin G; IgM: immunoglobulin M; IgA: immunoglobulin A; C3: complement 3; C4: complement 4; RF: rheumatoid factor; CCP: cyclic citrullinated peptide; ANA: antinuclear antibody; IL-2: interleukin-2; HBs: hepatitis B surface; HBc: hepatitis B core; HCV: hepatitis C virus; HTLV: human T cell leukemia virus; ATLV: adult T cell leukemia virus; EBV: Epstein–Barr virus; EA: early antigen; VCA: viral capsid antigen; EBNA: Epstein–Barr nuclear antigen; WT1: Wilms tumor 1

Laboratory test	Result	Reference range
CBC		
WBC count	19,100	2,700–8,800 /μL
Band neutrophils	2.0	0.0 - 6.0%
Segmented neutrophils	11.5	32.0–73.0%
Eosinophils	0.0	0.7–8.9%
Basophils	0.5	0.2–1.8%
Lymphocytes	43.5	16.5–47.0%
Atypical lymphocytes	37.0	0%
Monocytes	5.5	4.6–11.8%
RBC count	437	370–540 /μL
Hemoglobin	14.3	11.0–17.0 g/dL
Hematocrit	43.0	34.0–49.0 %
Platelets	20.9	14–34 × 10^4^/μL
Coagulation		
PT-INR	1.05	0.85-1.15
APTT	31.5	25.0–35.0 sec
D-dimer	4.93	<0.8 μg/mL
Biochemistry		
Total protein	6.9	6.6–8.2 g/dL
Albumin	3.7	3.9–4.9 g/dL
AST	36	8–38 U/L
ALT	46	4–44 U/L
LDH	454	124–212 U/L
g-GTP	49	16–73 U/L
Alkaline phosphatase	80	38–113 U/L
Total bilirubin	0.54	0.2–1.2 mg/dL
BUN	13.7	8.0–22.6 mg/dL
Creatinine	1.02	0.6–1.1 mg/dL
CK	59	56–244 U/L
ESR	17.0	<15 mm/hour
CRP	0.5	<0.3 mg/dL
Urinalysis		
Proteinuria	(1)	(-)
WBC	<1–4	<1–4 /HPF
RBC	<1–4	<1–4 /HPF
Casts	(-)	(-)/ HPF
Immunological		
IgG	1,463	870–1,700 mg/dL
IgA	383	110–410 mg/dL
IgM	154	35–220 mg/dL
C3	130	65–135 mg/dL
C4	22	13–35 mg/dL
RF	245.4	<15 IU/mL
Anti-CCP antibodies	12.3	<4.5 U/mL
ANA	<1:40	<1:40
Soluble IL-2 receptor	2,360.0	122–496 U/mL
β2-microglobulin	6.37	0.64–4.56 mg/L
Virological		
HBs-antigen	<0.05	<0.05 IU/mL
HBs-antibody	<10.0	<10.0 IU/mL
HBc-antibody	<1.0	<1.0 S/CO
HCV-antibody	<1.0	<1.0 COI
HTLV-I (ATLV)-antibody	Negative	Negative
EBV anti-EA IgG antibody	0.4	<0.5 index
EBV anti-VCA IgM antibody	1.3	<0.5 index
Anti-VCA IgG antibody	1:160	<1:10
Anti-EBNA antibody	1:40	<1:10
Whole blood EBV-DNA	4.90	<1.60 log IU/mL
Other		
Whole blood WT1 mRNA	<50	<50/μgRNA

**Figure 1:**
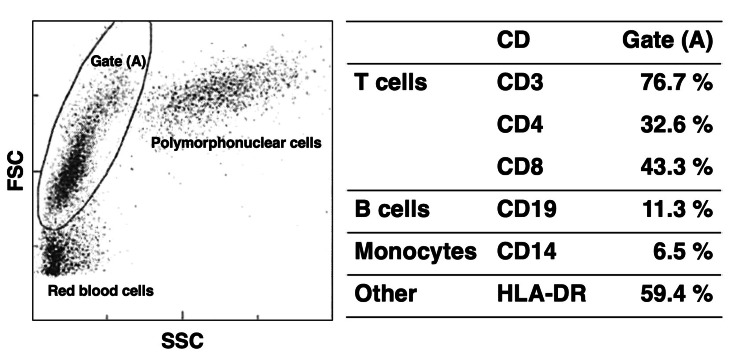
Flow cytometric analysis of peripheral blood mononuclear cells Flow cytometric analysis of peripheral blood mononuclear cells was performed on Day 0, the day the patient presented to the hospital with a two-week history of fever, night sweats, lymphadenopathy, and polyarthralgia. Gate A was set to encompass the region primarily containing lymphocytes and monocytes. Percentages for each cell subset were calculated based on the total number of cells within Gate A. HLA-D: human leukocyte antigen – DR isotype

Complete blood cell counts showed leukocytosis (19,100/μL), with lymphocytes comprising 43.5% (8,308/μL) of white blood cells and atypical lymphocytes accounting for 37% (7,067/μL). Biochemical testing revealed elevated serum lactate dehydrogenase (454 U/L; reference range, 124-222) and soluble interleukin-2 receptor (2,360 U/mL; reference range, 157-474), both indicating lymphocyte activation. C-reactive protein was slightly elevated (0.5 mg/dL; reference range, <0.14). Serological testing showed elevated rheumatoid factor (245.4 IU/mL; reference range, <15) and anti-CCP antibodies (12.3 U/mL; reference range, <4.5), while antinuclear antibodies were negative. EBV serology showed positivity for anti-VCA IgM antibodies (1.3; reference range, <0.5), anti-VCA IgG antibodies (1:160; reference range, <1:10), and anti-Epstein-Barr nuclear antigen (EBNA) IgG antibodies (1:40; reference range, <1:10). Whole blood EBV-DNA levels were elevated at 4.90 log IU/mL (approximately 79,433 IU/mL; lower limit of detection: 1.60 log IU/mL). As shown in Figure 1, flow cytometry of peripheral blood mononuclear cells demonstrated a CD4/CD8 ratio of 0.75, with CD8-positive T cell predominance. HLA-DR-positive cells accounted for 59.4%, exceeding the combined proportion of CD19+ B cells and CD14+ monocytes (17.8%).

**Figure 2:**
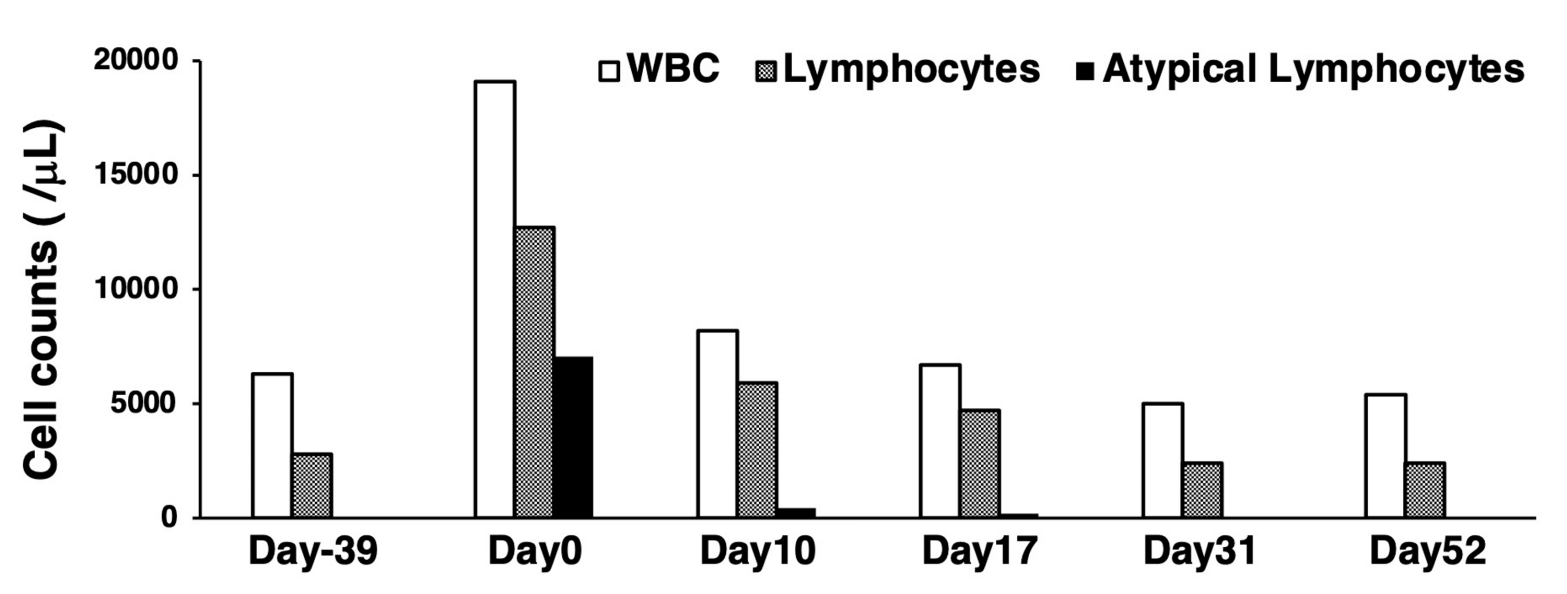
Trends in white blood cell (WBC), lymphocyte, and atypical lymphocyte counts over time Day 0 denotes the patient’s hospital presentation with a 2-week history of fever, night sweats, lymphadenopathy, and polyarthralgia attributed to Epstein–Barr virus reactivation. Day –39 represents a routine outpatient laboratory evaluation conducted 39 days prior to Day 0. White bars indicate total white blood cell counts, hatched bars indicate lymphocyte counts, and black bars indicate atypical lymphocyte counts.

Although dual staining with CD3 or CD8 and human leukocyte antigen (HLA)-DR was not performed, the findings suggested that the atypical lymphocytes were primarily activated CD8-positive T cells. Computed tomography (CT) revealed multiple enlarged lymph nodes (cervical, axillary, mediastinal, abdominal, and inguinal) and splenomegaly. Discontinuation of MTX and GLM led to rapid defervescence and resolution of lymphadenopathy, with disappearance of atypical lymphocytes (Figure 2). 

**Figure 3:**
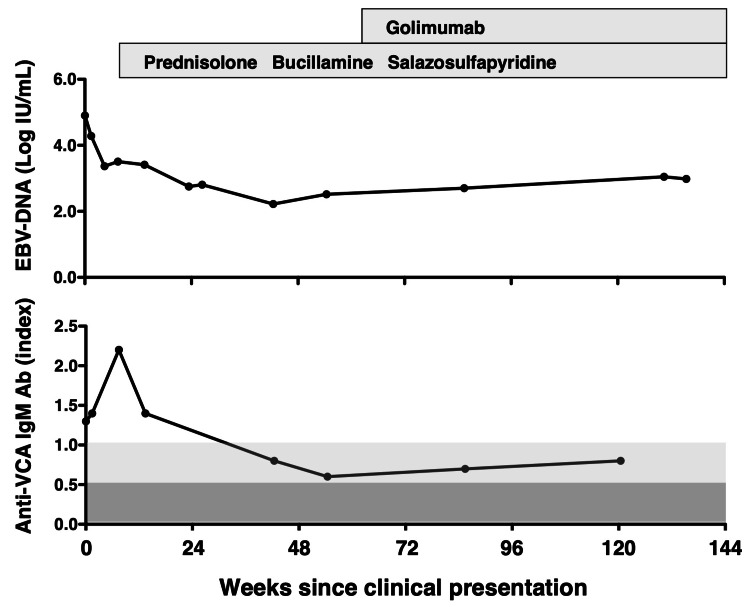
Longitudinal trends in Epstein–Barr virus (EBV)-DNA levels (log IU/mL; upper panel) and anti–viral capsid antigen (VCA) IgM antibody levels (index; lower panel) following clinical presentation of EBV reactivation. Week 0 denotes the week of hospital presentation with a two-week history of fever, night sweats, lymphadenopathy, and polyarthralgia attributed to EBV reactivation. The timing and duration of immunosuppressive therapies, including prednisolone, bucillamine, salazosulfapyridine, and golimumab, are indicated above the graphs. In the lower panel, background shading reflects anti–VCA IgM interpretation zones: dark gray (index < 0.5), negative (–); light gray (0.5 ≤ index < 1.0), equivocal (+/–); unshaded (index ≥ 1.0), positive (+). Ab: antibody.

While anti-VCA IgM titers declined, they remained detectable. Despite reductions in blood EBV-DNA and anti-VCA IgM antibody levels, both markers remained present (Figure 3), without recurrence of clinical symptoms.

Approximately eight weeks after discontinuation of MTX and GLM, the patient experienced a clinical relapse of RA, presenting with recurrence of joint symptoms requiring treatment. He was subsequently treated with conventional synthetic DMARDs, including bucillamine (200 mg/day) and salazosulfapyridine (1,000 mg/day), along with PSL (8 mg/day) and diclofenac (75 mg/day). This regimen allowed tapering of PSL to 4 mg/day over approximately 10 months; however, persistent joint symptoms indicated the need for therapeutic intensification and reconsideration of treatment strategy. Given the patient’s prior sustained clinical response to GLM in combination with MTX, GLM was reintroduced at 50 mg/month as monotherapy with close monitoring in light of the prior EBV-related adverse events, leading to clinical remission and allowing tapering of PSL from 4 mg/day to 3 mg/day. Remission was sustained under continued low-dose glucocorticoid therapy and monthly GLM administration, without recurrence of fever, lymphadenopathy, or other symptoms indicative of EBV reactivation. Serial monitoring showed that peripheral lymphocyte counts remained within normal ranges, and no atypical lymphocytes were detected, and that blood EBV-DNA levels remained stable throughout the 18-month post-reintroduction period. Notably, anti-VCA IgM antibodies remained positive, albeit at low levels, throughout the observation period (Figure 3).　

## Discussion

This case illustrates a rare clinical scenario in which an anti-TNF agent was successfully reintroduced in a patient with RA and persistent EBV reactivation. The patient experienced symptomatic EBV reactivation while receiving MTX and GLM, presenting with fever, lymphadenopathy, and atypical lymphocytosis, along with a marked elevation in EBV-DNA levels and persistent anti-VCA IgM seropositivity. Although clinical symptoms resolved promptly after discontinuing MTX and GLM, EBV-DNA and anti-VCA IgM remained detectable, suggesting a sustained early humoral response or delayed resolution of the primary immune reaction in the absence of overt disease. Notably, GLM was reintroduced during ongoing EBV reactivation, leading to restored disease control without recurrence of EBV-related symptoms. To our knowledge, this is the first documented case of successful reintroduction of an anti-TNF agent in a patient with RA amid prolonged EBV reactivation and sustained IgM seropositivity.

The prolonged elevation of EBV-DNA resembled, though did not meet, the diagnostic criteria for CHEBV, typically defined in pediatric transplant recipients as whole-blood EBV-DNA levels ≥10,000 IU/mL persisting for >6 months [8]. Among adult patients with autoimmune diseases receiving immunosuppressive therapy, no standardized definition or threshold for CHEBV exists. Differential diagnoses considered included infectious mononucleosis, CAEBV, and MTX-LPD, a condition in which EBV often plays a pathogenic role. Infectious mononucleosis was deemed unlikely, given that EBNA IgG positivity suggested past rather than primary infection [5]. CAEBV was similarly ruled out due to the absence of sustained mononucleosis-like symptoms, such as prolonged fever or hepatosplenomegaly, and the rapid resolution of atypical lymphocytosis after cessation of immunosuppressive therapy [14]. Since clonality testing and lymph node biopsy were not conducted, the possibility of MTX-LPD cannot be excluded. However, a marked increase in peripheral atypical lymphocytes, as observed in this case, is uncommon in MTX-LPD, which more typically presents with lymphadenopathy or extranodal mass-forming lesions [4,15]. Therefore, this case is considered to represent a CHEBV-like condition arising under immunosuppression but following a benign clinical course without progression to MTX-LPD. In future cases with similar presentations, clonality assessment will be crucial for distinguishing between reactive and neoplastic processes.

The persistent anti-VCA IgM antibody observed in this patient is of particular clinical interest. Although anti-VCA IgM typically appears transiently during primary EBV infection and declines with IgG class switching [5], its continued detection likely reflects ongoing antigenic stimulation from reactivated EBV. Notably, similar persistence of IgM antibodies has been described in cytomegalovirus infection, particularly under immunosuppressive conditions, and may reflect chronic antigen exposure or impaired immune regulation. While less well-characterized in EBV, a parallel mechanism may be at play in this case. On the other hand, it cannot be precluded that immune dysregulation associated with RA may have contributed to the persistence of IgM responses, possibly through mechanisms such as polyclonal B cell activation or impaired immune tolerance [16,17]. This is exemplified by the frequent co-expression of IgM and IgG isotypes of rheumatoid factor and anti-cyclic citrullinated peptide antibodies in RA [18,19], and similar patterns of isotype persistence are observed in other autoimmune diseases such as systemic lupus erythematosus [20]. Although the mechanisms remain unclear, they may involve chronic antigenic stimulation by persistent autoantigens or intrinsic defects in immune regulation that allow the simultaneous maintenance of early-phase (IgM) and class-switched (IgG) responses. This interpretation, however, remains speculative and warrants further immunological investigation.

The management of autoimmune diseases such as RA in the context of EBV reactivation during immunosuppressive therapy remains a complex and unresolved clinical issue. Immunosuppressive agents, including MTX, TNF inhibitors, and JAK inhibitors, can impair cytotoxic T cell-mediated immune surveillance, predisposing susceptible individuals to viral reactivation. Unlike the transplant setting, where EBV-DNA-guided preemptive strategies are well established [6], there is no approved antiviral therapy for EBV and no universally accepted guidelines or threshold values for interpreting EBV-DNA levels in patients with autoimmune diseases. Consequently, treatment must be individualized, taking into account clinical presentation, immunosuppressive burden, and trends in EBV-DNA levels and serologic markers. Although consensus-based thresholds have not been defined for RA, insights from transplantation may provide useful benchmarks. In HCT and SOT, EBV-DNA levels ranging from 1,000 to 10,000 IU/mL are often used to initiate preemptive measures to prevent post-transplant LPD. In pediatric liver transplant recipients, Shizuku et al. proposed defining CHEBV as EBV-DNA levels ≥10,000 IU/mL persisting for >6 months and recommended tapering immunosuppressants, such as calcineurin inhibitors, once levels exceed 5,000 IU/mL [8]. Their findings showed that many patients could be managed without antiviral therapy through immunosuppression adjustment alone. While RA differs from transplantation in pathophysiology and immune status, the concept of viral load-based risk stratification may be cautiously applied to autoimmune settings. Future studies should aim to define disease-specific EBV-DNA thresholds and monitoring strategies to enhance patient safety and mitigate EBV-associated complications. 

Recent pathological findings from a study on RA-associated OIIA-LPD by Hoshida et al. showed that EBER-1-positive lesions were significantly more frequent in patients receiving both MTX and TNF inhibitors than in those on MTX monotherapy [4], suggesting that combined immunosuppression may elevate the risk for EBV-associated LPD. The current case, involving EBV reactivation during concurrent MTX and GLM therapy, exemplifies this clinical scenario and underscores the need for vigilant EBV monitoring in patients receiving combination immunosuppressive regimens, especially when considering continuation or reinitiation of biologic agents. Although this case did not fulfil the diagnostic criteria for RA-associated OIIA-LPD, the presence of EBV reactivation and transient lymphoproliferative features necessitated careful therapeutic consideration. Given that rituximab is not approved for RA in Japan and that the patient had previously responded well to GLM, albeit in combination with MTX, this agent was reintroduced under close monitoring. IL-6 receptor inhibitors were retained as a secondary option and would have been initiated if GLM failed to control disease activity or in the event of EBV-related relapse.

This case report has several important limitations. First, persistent anti-VCA IgM antibodies may not necessarily indicate continuous EBV reactivation; they could reflect nonspecific polyclonal B cell activation commonly seen in chronic inflammatory states such as RA. Although Western blot analysis can help distinguish specific anti-EBV IgM responses from nonspecific reactivity, this was not performed in the present case. Second, EBV-LPD-including MTX-LPD-could not be definitively excluded, given the absence of tissue-based assessment. While the clinical course appeared benign, the lack of histopathological confirmation is a diagnostic limitation. Third, because this case involved GLM specifically, generalizability to other TNF inhibitors remains uncertain. Caution is advised in extrapolating these findings across the therapeutic class. Finally, although no clinical relapse or adverse events occurred during the 18-month follow-up after GLM reintroduction, this period may not be sufficient to exclude late-onset complications. Ongoing monitoring is essential to assess the long-term safety of resuming immunosuppressive therapy in such contexts.

## Conclusions

This case suggests that GLM, a TNF-α inhibitor, may be safely reintroduced in patients with RA and prolonged EBV reactivation, as indicated by persistent EBV-DNA and anti-VCA IgM positivity. The absence of clinical relapse supports the potential feasibility of this approach under careful surveillance. This case underscores the need for individualized management strategies and risk-based monitoring in patients with EBV-specific reactivation, particularly in the absence of disease-specific viral load thresholds or standardized management protocols. Notably, this reflects a significant disparity in the availability of EBV monitoring strategies, transplant medicine benefiting from well-established guidelines, while autoimmune conditions remain without comparable standards. Further accumulation of comparable cases will be critical to refining risk stratification models and informing prospective studies aimed at establishing evidence-based guidelines for managing immunosuppressive therapy in EBV-DNA-positive individuals with autoimmune disease.
